# Chronic *Trypanosoma cruzi* infection potentiates adipose tissue macrophage polarization toward an anti-inflammatory M2 phenotype and contributes to diabetes progression in a diet-induced obesity model

**DOI:** 10.18632/oncotarget.7630

**Published:** 2016-02-23

**Authors:** María E. Cabalén, María F. Cabral, Liliana M. Sanmarco, Marta C. Andrada, Luisina I. Onofrio, Nicolás E. Ponce, María P. Aoki, Susana Gea, Roxana C. Cano

**Affiliations:** ^1^ Facultad de Ciencias Químicas, UA Área CS. AGR. ING. BIO Y S CONICET. Universidad Católica de Córdoba, Córdoba, Argentina; ^2^ Centro de Investigaciones en Bioquímica Clínica e Inmunología (CIBICI-CONICET), Córdoba, Argentina

**Keywords:** obesity, immunometabolism, adipose tissue macrophages, innate immunity, Trypanosoma cruzi, Immunology and Microbiology Section, Immune response, Immunity

## Abstract

Chronic obesity and Chagas disease (caused by the protozoan *Trypanosoma cruzi*) represent serious public health concerns. The interrelation between parasite infection, adipose tissue, immune system and metabolism in an obesogenic context, has not been entirely explored. A novel diet-induced obesity model (DIO) was developed in C57BL/6 wild type mice to examine the effect of chronic infection (DIO+I) on metabolic parameters and on obesity-related disorders. Dyslipidemia, hyperleptinemia, and cardiac/hepatic steatosis were strongly developed in DIO mice. Strikingly, although these metabolic alterations were collectively improved by infection, plasmatic apoB100 levels remain significantly increased in DIO+I, suggesting the presence of pro-atherogenic small and dense LDL particles. Moreover, acute insulin resistance followed by chronic hyperglycemia with hypoinsulinemia was found, evidencing an infection-related-diabetes progression. These lipid and glucose metabolic changes seemed to be highly dependent on TLR4 expression since TLR4−/− mice were protected from obesity and its complications. Notably, chronic infection promoted a strong increase in MCP-1 producing macrophages with a M2 (F4/80+CD11c-CD206+) phenotype associated to oxidative stress in visceral adipose tissue of DIO+I mice. Importantly, infection reduced lipid content but intensified inflammatory infiltrates in target tissues. Thus, parasite persistence in an obesogenic environment and the resulting host immunometabolic dysregulation may contribute to diabetes/atherosclerosis progression.

## INTRODUCTION

The recognition of obesity as a main source of human disease including type 2 diabetes (T2D) and cardiovascular disorders, has provoked fierce interest in metabolic dysfunction, and identified inflammatory insulin resistance (IR) as its central pathophysiological mechanism [1]. Involvement of toll-like receptor 4 (TLR4) signaling in the pathogenesis of metabolic disorders has been well documented [2, 3]. The current literature demonstrates that TLR4 deficiency protects from IR [4] and reduces adipose tissue (AT) and vascular inflammation as well as hepatic steatosis after a high-fat diet (HFD) [4, 5]. Prominently, pathogenic responses to obesity have been ascribed to AT dysfunction that promotes the secretion of a large panel of cytokines and chemokines, and leads to the initiation of pro-inflammatory events, oxidative stress and tissue dysfunction [6]. This process involves the coordinated interaction of various cell populations comprising the AT stromal vascular fraction (SVF) including AT macrophages (ATMs), which have received a great deal of attention due to their progressive accumulation during tissue expansion [5, 7]. Many studies have sought to categorize ATMs according to the M1/M2 macrophage system [8]. Although the general consensus in the field is that obesity induces the polarization from M2 into M1 phenotype [7], several lines of evidence have pointed to a predominant and dynamic M2 polarization of ATMs during obesity in mice and humans [7, 9-11].

Overall, many studies demonstrating the negative impact of excess adiposity on immune function have focused on the study of artificial metabolic extreme models. Although these approaches yield valuable insight into human disease, they are limited to describe the non-extreme forms of obesity-metabolic dysfunction that dominate the clinical field [1].

Chagas disease is a vector-borne parasitic disease caused by infection with the protozoan *Trypanosoma cruzi*. It is estimated that 10 million people are infected worldwide and more than 25 million people are at risk of infection [12]. In the past decades, mainly as a result of increased population migrations, the diagnosed cases have increased also in non-endemic countries [13]. Parasite persistence eventually results in severe complications in the cardiac and gastrointestinal tissues, but it also affects the reticuloendothelial system as the liver, spleen, and bone marrow. It is now well established that AT is also an important reservoir for the parasite [14, 15]. Recently, we demonstrated that *T. cruzi* infection is a potent risk factor for non-alcoholic steatohepatitis [16], which is clearly associated to obesity-metabolic consequences.

Even though there is enough evidence of the influx of macrophages into AT during the early stage of infection [15], the effect of *T. cruzi* infection on ATMs polarization under a chronic obesogenic nutritional challenge has been poorly explored. Mechanistically, CD36 scavenger receptor has been involved in the development and progression of obesity and its cardiometabolic Separate complication to [17]. However, the effect of chronic *T. cruzi* infection on CD36 expression in VAT and how parasites could modulate host metabolism and inflammation remains to be elucidated.

Accordingly, the purpose of this study was to develop a chronic and non-extreme diet-induced obesity DIO model associated to T2D and cardiovascular disorders progression. We evaluated changes in morphometric parameters such as expansion of VAT, metabolic homeostasis, and systemic inflammation, as well as the role of TLR4 signaling in this metabolic context. Particularly on VAT, ATMs polarization and CD36 receptor expression were further analyzed to understand chronic host-parasite interaction in an obesity and Chagas disease scenario.

## RESULTS

### Morphometric and metabolic parameters in a diet-induced obesity model and the effect of chronic *Trypanosoma cruzi* infection

**Figure 1:**
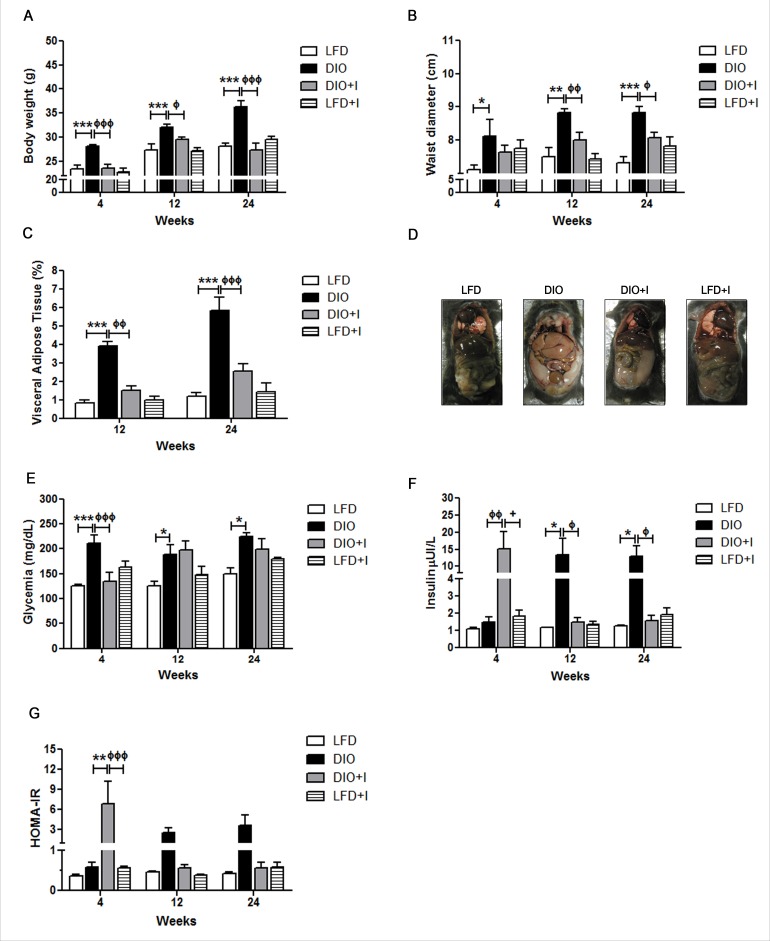
Changes in morphometric and metabolic parameters associated to a diet-induced obesity model and *T. cruzi* infection All parameters were registered at 4, 12 and 24 weeks in mice from LFD, DIO, DIO+I and LFD+I groups. Body mass abnormalities were analyzed by **A.** body weight in grams, **B.** waist diameter in centimeters, and **C.** visceral adipose tissue expansion expressed as the percentage of tissue weight in relation to total body weight. **D.** Representative image of a mouse from each group at 24 weeks. Determinations of plasma levels of **E.** glucose, **F.** insulin, and **G.** HOMA-IR were made. Data are shown as mean ± SEM of ten mice per group from one experiment representative of two performed. Significance of differences using two-way ANOVA is indicated as follows: DIO *vs*. LFD: ^***^ *p* < 0.001, ** *p* < 0.01, * *p* < 0.05. DIO *vs*. DIO+I: ^ффф^ *p* < 0.001, ^фф^ *p* < 0.01, ^ф^ *p* < 0.05.

To determinate whether medium fat diet (MFD), low fructose and a minimal streptozotocin (STZ) dose could develop a non-drastic DIO model, we first assessed the variation of morphometric parameters in DIO group compared to low fat diet (LFD) group for 24 weeks. We observed a time-dependent increase in body weight and waist diameter in DIO group (Figure 1A-1B). Additionally, we evaluated the increment of VAT and found that it was significantly higher in DIO than in LFD group at 12 and 24 weeks (Figure 1C). Interestingly, the effect of *T. cruzi* infection in this DIO model (DIO+I) showed a significant reduction in body weight, waist diameter and VAT content compared to DIO group (Figure 1A-1D), and no significant differences were seen between DIO+I and LFD+I groups.

Furthermore, DIO group showed hyperglycemia from 4 to 24 weeks associated to hyperinsulinemia and peripheral IR (increase in HOMA-IR) at 12 and 24 weeks (Figure 1E-1G). Conversely, DIO+I displayed a significant reduction of glycemia during the acute phase of infection (at 4 weeks) in relation to DIO group, whereas similar glucose levels were reached at 12 and 24 weeks (Figure 1E). Interestingly, acute IR was observed in DIO+I group at 4 weeks, but lately hyperglycemia concomitantly with both, low HOMA-IR index and hypoinsulinemia were observed, suggesting a progression to a *T. cruzi* infection-related-diabetes.

### The interaction between diet and *T. cruzi* infection enhances the development of cardiovascular disorders

**Figure 2:**
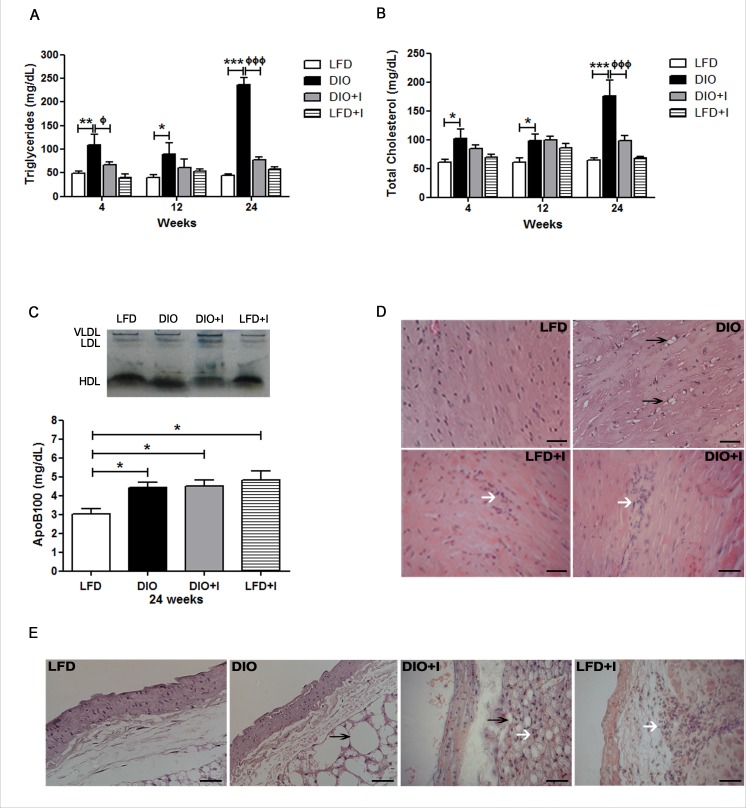
The interaction between diet and *T. cruzi* infection enhances the development of cardiovascular disorders Lipid metabolism was analyzed by the measurements of plasma levels of **A.** triglycerides and **B.** total cholesterol after 12h fasting in mice from either LFD, DIO, DIO+I or LFD+I groups. **C.** Lipoprotein pattern was assessed by electrophoresis. ApoB100 was quantified at 24 weeks and analyzed by one-way ANOVA: DIO, DIO+I and LFD+I *vs*. LFD: * *p* < 0.05. **D.** A representative hematoxylin and eosin image of heart tissue from each group is shown (400x). The arrows represent inflammatory foci (white) and lipid deposition (black). Scale bar = 20μm. **E.** A representative hematoxylin and eosin image of abdominal aorta from each group is shown (400x). The arrows represent inflammatory foci (white) and lipid deposition (black). Data are shown as mean ± SEM of seven mice per group from one experiment representative of two performed. Statistical analysis was made by using two-way ANOVA test as follows: DIO *vs*. LFD: ^***^ *p* < 0.001, ** *p* < 0.01, * *p* < 0.05, and DIO *vs*. DIO+I: ^ф^ *p* < 0.05, ^ффф^ *p* < 0.001.

The risk of developing cardiovascular alterations was assessed by determining plasma triglycerides (TG) and total cholesterol (TC) levels together with a lipoprotein electrophoretic analysis. The highest levels of TG and TC were demonstrated in DIO group at 24 weeks compared to LFD and DIO+I groups. Interestingly, although DIO+I improved dyslipidemia compared to DIO group (24 weeks), plasmatic apoB100 levels were significantly increased in all groups compared to LFD, suggesting the presence of pro-atherogenic small and dense LDL particles (Figure 2A-2C).

In addition, heart tissue from DIO group showed ectopic lipid accumulation and a slight inflammatory cell infiltration at 12 weeks (Figure 2D). Conversely, DIO+I group revealed a minor lipid deposition with an increased inflammatory cell infiltration. Amastigote nests were not observed at 12 and 24 weeks, findings which are clearly compatible with a chronic Chagas myocardiopathy (Figure 2D). Likewise, abdominal aorta images in DIO group showed disturbances in the epithelium architecture with the presence of hypertrophied adventitial adipocytes, whereas these type of alterations were not observed in DIO+I group (Figure 2E). However, an increased number of infiltrating cells on the aorta's adventitia was found in this infected group (Figure 2E).

**Figure 3:**
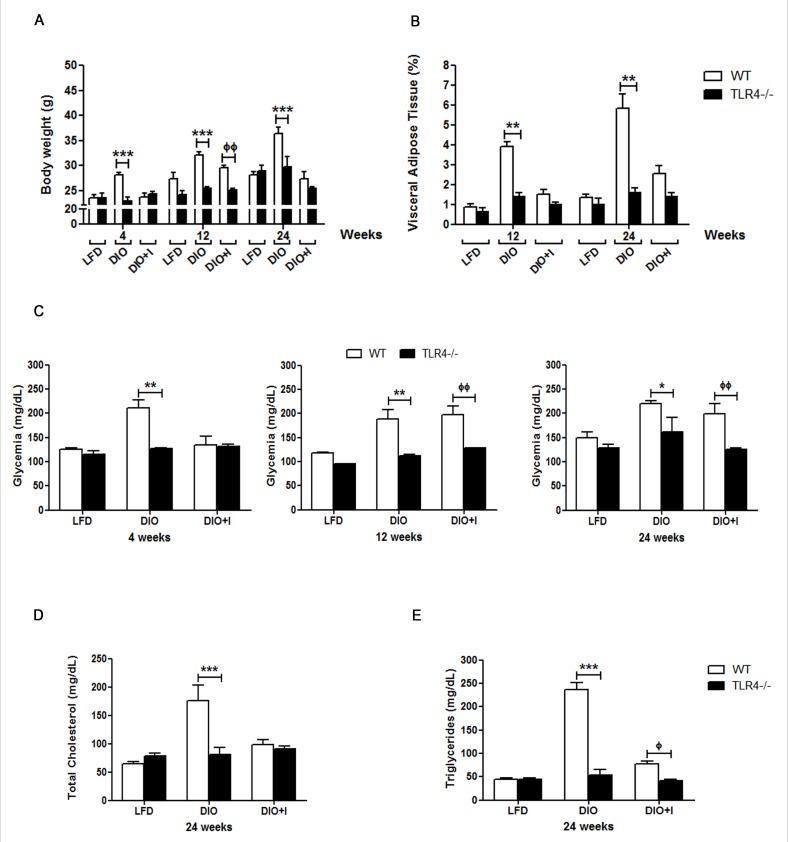
TLR4 signaling mediates obesity development, glucose and lipid abnormalities triggered by diet and *T. cruzi* infection in this obesity model **A.** Body weight, **B.** percentage of visceral adipose tissue, **C.** glycemia, **D.** total cholesterol and **E.** triglycerides plasma levels were compared between WT and TLR4−/− groups. *n* = seven mice per group. Data are shown as mean ± SEM. Statistical studies were performed using two-way ANOVA test. DIO *vs*. TLR4 −/− DIO: ^***^ *p* < 0.001, ** *p* < 0.01, * *p* < 0.05. DIO+I *vs*. TLR4 −/− DIO+I: ^фф^ *p* < 0.01.

With the aim of studying the role of TLR4 signaling in our model, several metabolic parameters were measured in TLR4-deficient (TLR4−/−) mice. TLR4−/− DIO mice showed a significant reduction in body weight compared to WT from 4 to 24 weeks, while no significant changes were observed between TLR4−/− DIO and TLR4−/− LFD groups (Figure 3A). Importantly, VAT percentage was significantly decreased in TLR4−/− DIO group in relation to WT group at 12 and 24 weeks (Figure 3B). In addition, a significant decrease in glycemia was observed in TLR4−/− DIO when compared to WT mice. A similar behavior was seen between TLR4−/− DIO+I and WT mice (Figure 3C). Moreover, a significant reduction in TC and TG plasma levels was found on TLR4−/− DIO in comparison to WT group at 24 weeks (Figure 3D-3E). Data from TLR4−/− LFD+I mice were not included as they showed an increased susceptibility to *T. cruzi* infection and so, a high mortality during the acute phase of infection (Supplemental Figure 1).

Strikingly, a significant decrease in apoB100 levels was seen in TLR4−/−DIO and DIO+I in relation to their respective WT groups (Supplemental Figure 2).

### Diet and infection induce an imbalance of classical pro-inflammatory and leptin/adiponectin cytokines

**Figure 4:**
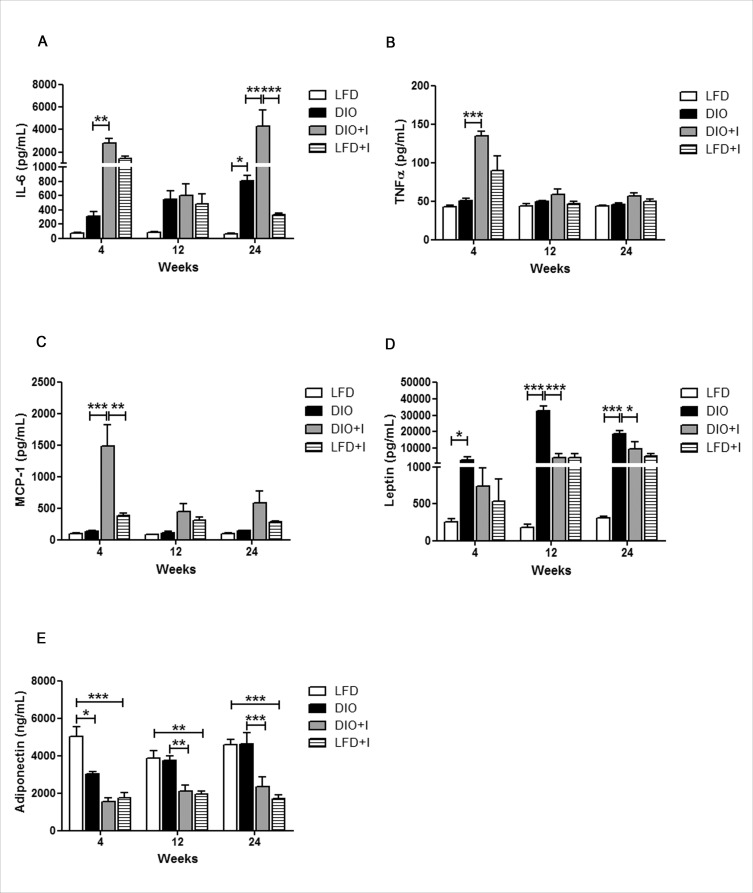
Diet and infection induce an imbalance of classical pro-inflammatory and leptin/adiponectin cytokines Cytokine levels were quantified by ELISA in plasma obtained at 4, 12 and 24 weeks from LFD, DIO, DIO+I and LFD+I groups: **A.** IL-6, **B.** TNF-α, **C.** MCP-1, **D.** leptin and **E.** adiponectin. Data are shown as mean ± SEM of seven mice per group. A *p*-value < 0.05 was considered significant using two-way ANOVA test: DIO *vs*. LFD and DIO *vs*. DIO+I: ^***^ *p* < 0.001, ** *p* < 0.01, * *p* < 0.05.

It has been well documented that chronic inflammation occurs in AT mainly due to macrophage infiltration during obesity, and this process leads to relevant changes in both, metabolic and inflammatory responses. In our DIO model, there was a significant increase in IL-6 plasma levels compared to LFD group only at 24 weeks. However, a significant increase in this cytokine was observed at 4 and 24 weeks in the DIO+I group, when compared to DIO group (Figure 4A). TNF-α showed a significant increase only at 4 weeks in DIO+I *vs*. DIO mice, while no significant changes were observed among other groups at the studied times (Figure 4B). Similarly, the highest systemic levels of MCP-1 were detected in DIO+I group at 4 weeks (Figure 4C).

Hyperleptinemia was found from 4 to 24 weeks in all experimental groups compared to LFD (Figure 4D) and remarkably, the highest levels were observed in DIO group at 12 and 24 weeks. Adiponectin was significantly decreased at 4 weeks in all groups compared to LFD group. However, its levels remained low only in the infected groups at 12 and 24 weeks (Figure 4E).

It is known that IL-6 and TNF-α cytokines are able to stimulate the production of acute phase proteins in liver during inflammation. The protein patterns revealed an increment in α2 macroglobulin fraction in DIO compared to LFD group at 4 weeks. Both, α1 and α2 fractions were highly increased in the infected groups compared to DIO and LFD groups. Concomitantly, an increase in the gamma globulin fraction was found in the infected groups (Supplemental Figure 3).

### *T. cruzi* infection induces a similar increase in CD36 receptor expression but results in an exacerbated inflammatory response on VAT in this obesity model

**Figure 5:**
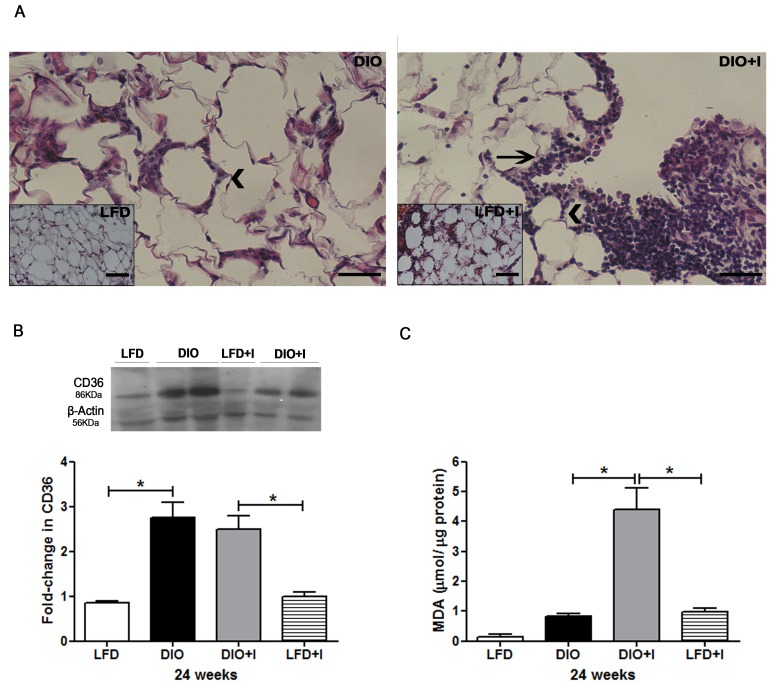
Diet induces an increased CD36 scavenger receptor expression, and *T. cruzi* infection exacerbates inflammatory cell infiltration and oxidative stress in VAT **A.** Representative hematoxylin and eosin images of VAT from DIO and DIO+I mice (400x). Inserts are from LFD (on the left) and LFD+I (on the right) mice. Arrowheads and black arrows represent CLSs and inflammatory foci, respectively. Scale bar = 40μm. **B.** Immunoblot of CD36 was performed on VAT lysates at 24 weeks. The quantitative analysis is shown and expressed as fold change in CD36 protein expression. Error bars represent SEM, * *p* < 0.05 (*n* = 4 per group). **C.** Extent of lipid peroxidation at 24 weeks was evaluated and lipid peroxides were expressed as μmol MDA/μg protein. Data are shown as mean ± SEM of six mice per group from one experiment representative of three performed. A *p*-value < 0.05 was considered significant using one-way ANOVA test.

Excessive VAT accumulation is a main feature of obesity development and this tissue represents an important reservoir for parasites. Our results showed a dysfunctional VAT with the presence of hypertrophied adipocytes and crown-like structures (CLSs) on DIO group (Figure 5A, on the left). Conversely, *T. cruzi* infection promoted a decrease in adipocyte size accompanied by an important increase in the number of infiltrating cells and CLSs, as observed in DIO+I group at 24 weeks (Figure 5A, on the right).

Interestingly, an increased CD36 protein expression in VAT was revealed in DIO and DIO+I compared to the respective control groups (Figure 5B).

It is widely known that oxidative stress is an important mechanism in the pathogenesis of obesity. Thus, we evaluated the extent of lipid peroxidation by thiobarbituric acid reactive substances (TBARs) in VAT; only DIO+I mice exhibited a significant increase in malonyldialdehyde (MDA) levels at 24 weeks (Figure 5C).

**Figure 6:**
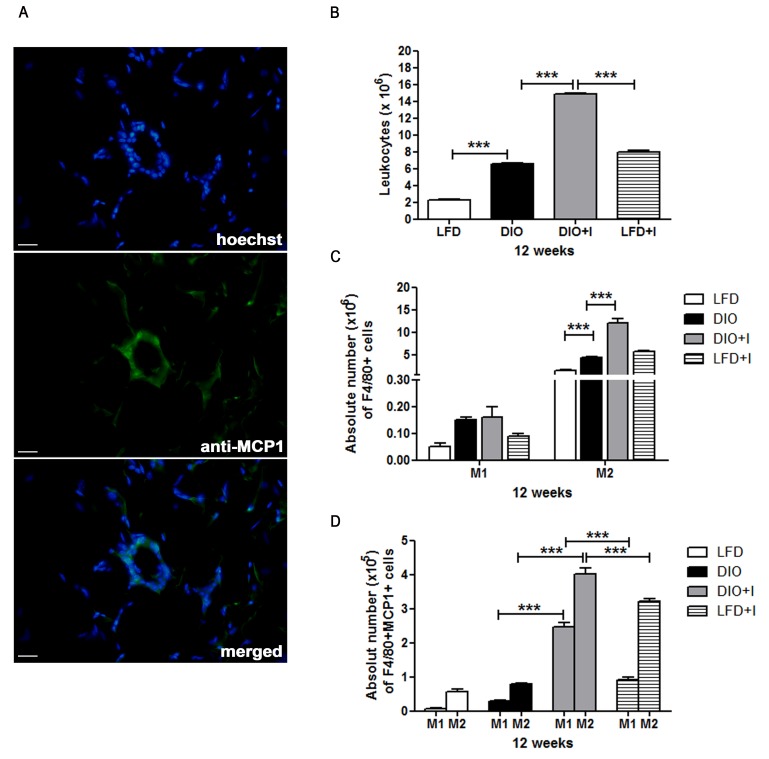
Increased number of M2- phenotype macrophages and its MCP-1 production are induced by diet and exacerbated by *T. cruzi* infection in VAT **A.** Representative images for MCP-1 detection (400x) of epididymal (visceral) AT from DIO+I mice at 12 weeks. Rabbit anti-mouse MCP-1 and anti-rabbit conjugated with FITC antibodies were used. Scale bar = 20 μm. **B.** Epididymal AT leukocytes from different groups of mice obtained at 12 weeks, and **C.** the absolute numbers of F4/80+CD11c+CD206- (M1) and F4/80+CD11c-CD206+ (M2) cells are shown. **D.** The absolute number of MCP-1 producing F4/80+ CD11c+ (M1) and F4/80+CD206+ (M2) cells of epididymal AT are shown. Assays were performed by flow cytometry and shown as mean ± SEM of six mice from one experiment representative of two performed: ^***^ *p* < 0.001.

Considering that MCP-1 is one of the key chemokines that regulate migration of monocytes/macrophages into inflamed tissues, we analyzed the local production of this chemokine on VAT from DIO+I group and showed that MCP-1 was mainly localized on the CLSs of this dysfunctional tissue (Figure 6A). In addition, a flow citometric analysis of leukocytes isolated from SVF was performed at 12 weeks in order to study which macrophage phenotype was the prevalent on our model. At this time, a significant increase in the percentage of local infiltrating leukocytes was observed in DIO, DIO+I and LFD+I compared to LFD control mice (Figure 6B) and interestingly, a synergism was observed on DIO+I group.

A prevalence of F4/80^+^CD11c^−^CD206^+^ (M2) over F4/80^+^CD11c^+^CD206^−^ (M1) macrophages was shown in all experimental groups (Figure 6C). Notably, the highest absolute number of ATMs with a M2 switch polarization was found in DIO+I group. Further, a higher MCP-1 production in M2 than in M1 macrophages was also observed in all groups (Figure 6D).

### Non-alcoholic fatty liver disease in diet- induced obesity is modulated by *T. cruzi* infection

**Figure 7:**
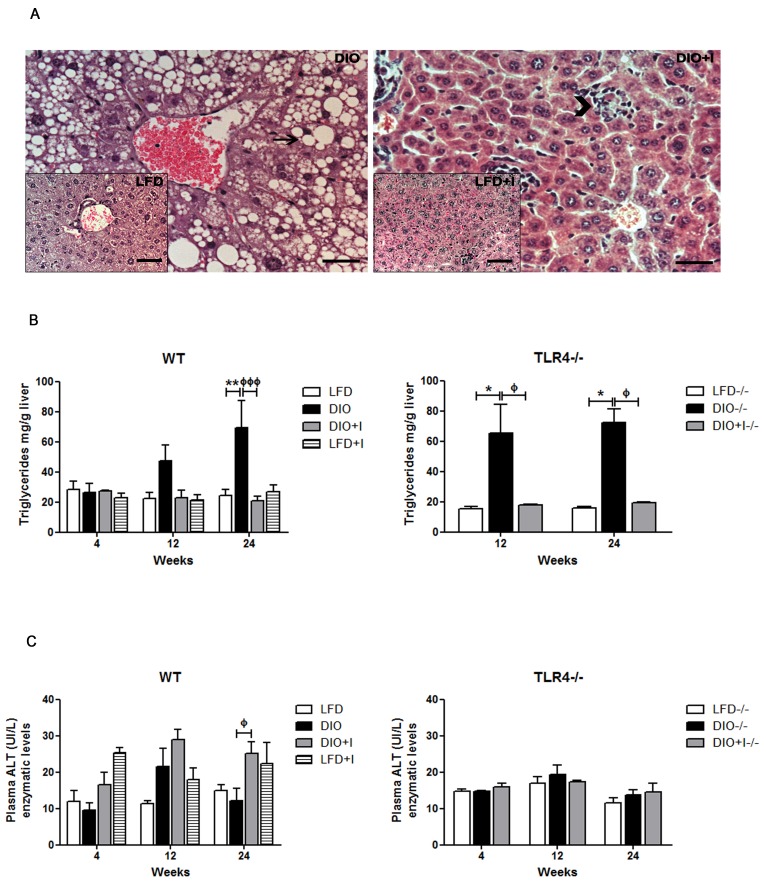
Non-alcoholic fatty liver disease in DIO model is modulated by *T. cruzi* infection **A.** Representative hematoxylin and eosin images of hepatic tissue from all groups (400x): DIO image with an insert for LFD group (on the left), DIO+I image with an insert for LFD+I group (on the right). Arrow represents lipid content and arrowhead, inflammatory foci. Scale bar = 40μm. **B.** Hepatic triglyceride content was determined for all groups from WT and TLR4−/−mice and expressed as mg/g of liver tissue. **C.** Plasma ALT enzymatic levels of WT and TLR4−/−groups. Results are representative of one experiment of two performed. Data are shown as mean ± SEM of seven mice per group. Significance of differences using two-way ANOVA is indicated as follows: DIO *vs*. LFD: ^**^ *p* < 0.01. DIO *vs*. DIO+I: ^ффф^ *p* < 0.001, ^ф^ *p* < 0.05. TLR4−/− DIO *vs*. LFD:* *p* < 0.05; TLR4−/− DIO *vs*. DIO+I: ^ф^*p* < 0.05.

At 12 weeks, the DIO model was able to induce the development of hepatic steatosis. Remarkably, a decrease in lipid deposition with an exacerbated local inflammatory response was detected in DIO+I compared to DIO group (Figure 7A). Supporting these results, we found a two- to three-fold increase of hepatic TG in WT DIO in relation to DIO+I group at 24 weeks (Figure 7B, on the left). While infection seemed to improve hepatic steatosis, alanine aminotransferase (ALT) enzymatic levels were significantly increased in plasma from WT DIO+I *vs*. DIO group at 24 weeks (Figure 7C, on the left). TLR4−/− mice showed no significant changes in ALT levels (Figure 7C, on the right). Interestingly, hepatic TG levels were significantly elevated only in TLR4−/− DIO group at 12 and 24 weeks, while in DIO+I, these levels were comparable to those of the LFD mice (Figure 7B, on the right).

### Diet leads to an increased parasitemia, myocardial inflammatory infiltrate and parasite load during acute *T. cruzi* infection

**Figure 8:**
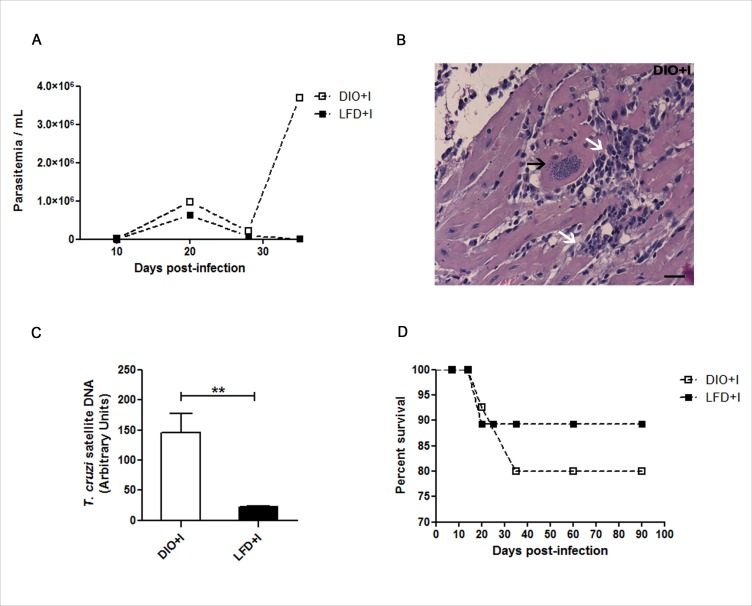
Diet leads to an increased parasitemia, myocardial inflammatory infiltrate and parasite load during acute *T. cruzi* infection **A.** Parasitemias were determined at different days after *T. cruzi* infection in DIO+I and LFD+I mice, *n* = seven mice per group. **B.** A representative hematoxylin and eosin heart image from DIO+I mouse at 35 days post infection is shown (400x). Black arrow indicates an amastigote nest and white arrows indicate inflammatory cell-infiltrations. Scale bar = 20μm. **C.** Quantitative assessment of the parasite load by q-PCR in hearts of the infected groups. Parasite quantification in DNA samples was performed amplifying a *T. cruzi* satellite sequence at 4 weeks. The relative load of parasites was normalized against the GADPH housekeeping gene control. Data are shown as mean ± SEM of four mice per group from one experiment representative of two performed. **p < 0.01 using Student's *t* test for comparison between infected groups. **D.** Percentage of survival from infected LFD and DIO groups.

Our results revealed that parasitemia was higher in DIO+I than in LDF+I group, reaching a first parasitemia peak at 20 days post-infection. Some animals from DIO+I group showed a second peak at 35 days, just before they finally died (Figure 8A). Remarkably, detectable amastigote nests with strong myocardial inflammatory infiltrates were seen in this group at 35 days (Figure 8B). Supporting these observations, the cardiac parasite load measured at 4 weeks revealed significantly higher levels of parasite DNA in DIO+l in comparison to LFD+I mice (Figure 8C). Also, a minor percentage of survival was found in DIO+I group up to 90 days post infection (Figure 8D).

## DISCUSSION

Obesity continues to grow worldwide, making it imperative that animal models sharing characteristics of human obesity and its co-morbidities be developed in the search for innovative treatments [18]. The cluster of metabolic alterations accompanying obesity has been extensively studied using HFD or genetic mouse models [1]. Metabolic disorders have been observed in humans and animals with high daily fructose (15-30%) intakes, but whether fructose consumed in a low amount has deleterious effects, is still unknown [19]. There is evidence supporting the role of high doses of STZ as an inductor of experimental T1D model [20]. We demonstrate for the first time that the mere feeding with a MFD and a low fructose concentration, combined with a minimal dose of STZ, lead to the development of a novel non-extreme DIO model. Our findings showed the induction of IR and progression to T2D, dyslipidemia, hyperleptinemia, cardiovascular abnormalities and hepatic steatosis. This model displayed a time-dependent increase in body weight attributable to diet composition, since no difference on food intake was appreciated between LFD and MFD (data not shown). On diet group, dyslipidemia and hyperglycemia associated to high insulin levels and an increase in the percentage of VAT and hepatic TG deposition were observed at 12 and 24 weeks. In addition, insulin and leptin resistance states were clearly developed, as described in other DIO and genetic models [21, 22]. It is known that a high flux of fructose to the liver perturbs glucose metabolism and glucose uptake pathways, leading to an enhancement of *de novo* TG synthesis which underlies the induction of IR [19, 23].

Interestingly, acute parasite infection in DIO+I induced a high insulin secretion which was able to maintain near to normal glucose levels, but during the chronic phase of infection, hyperglycemia was accompanied by low insulin levels, which may result in a progression to diabetes. In this regard, Nagajyothi *et al*. (2013) demonstrated that *T. cruzi* infection (Brazil strain) induced pancreatic inflammation and β-cell parasitism, which resulted in low insulin levels during the acute and chronic phase of infection [24]. Discrepancies observed in the acute phase could be attributed to differences on the assayed experimental conditions.

Recently, a clear mechanistic link has been demonstrated between pancreatic β-cell dysfunction and inflammation *via* the FFA-TLR4/MyD88 pathway [25, 26]. Remarkably, we reported that the lack of TLR4 signaling improves glucose and lipid metabolism, and protected from obesity development and premature atherosclerosis. However, its absence did not protect from lipid deposition in liver. We speculate that fructose could be responsible for this phenomenon since it is a key contributing factor for the synthesis of *de novo* TG [19], and TLR4 signaling may not be involved in mediating this effect.

Cardiovascular histomorphologic abnormalities observed in DIO mice were modified by parasite infection [27, 28]. *T. cruzi* infection induced a reduction on tissue lipid content together with an increased cell infiltration. The activation of host and/or *T. cruzi* lipases could be involved in tissue lipid reduction [15, 24, 29-31]. Additionally, chronic infection improved TG and TC plasma levels compared to DIO mice, in accordance to that reported by Nagajyothi *et al*. (2014) during the acute phase of infection in a HFD (60%) model [29]. This improvement could be due to the fact that this protozoan parasite has an incomplete de *novo* lipid metabolic machinery and needs to scavenge host lipids for its growth [30].

Considering that inflammation, hypercholesterolemia, and infection are pro-atherogenic risk factors, we evaluated apoB100 levels together with a systemic lipoprotein pattern analysis. Strikingly, the abnormal apoB100/lipoprotein pattern observed in DIO, DIO+I and LFD+I mice, suggests that diet and infection themselves could be able to contribute to an early pro-atherogenic state [32-35]. In line with our observations, it is known that *T. cruzi* has a high affinity for host lipoproteins/cholesterol and it uses lipids and LDL receptor for cell invasion [34].

We reported that parasite infection under an obesogenic diet context resulted in a strong systemic and local inflammatory response. This could probably explain the acute IR, tissue lipolysis, and body weight loss found in this study. Thus, we observed an intense acute inflammatory response produced by the increase in acute phase proteins and a wide range of plasma cytokines as TNF-α, IL-6 and MCP-1 in DIO+I group, as demonstrated in other reports [15, 29]. During chronic infection, this systemic inflammatory milieu was also encountered but to a lesser extent. Although *T. cruzi* infection improved leptin resistance in this DIO model, this adipokine remain elevated in relation to control groups. Leptin alterations in the acute phase of infection had been already reported by other authors [29, 36]. In line, *T. cruzi* invades the brain, affecting the central nervous system endocrine pathways, which may perturb insulin [24] and leptin secretion.

Consistent with other reports, adiponectin levels were reduced during acute parasite infection [36]. Our results provide the first evidence in demonstrating a sustained decrease in adiponectin levels during the chronic phase of infection,which could be considered as an additional cardiovascular risk factor for DIO+I and LFD+I groups [37]. Interestingly, an intense myocardial inflammatory process with a concomitant high parasite load was observed in DIO+I mice during the acute phase of infection. These findings could be responsible, at least in part, for the development of the more severe acute/chronic myocardiopathy reported in this group.

Visceral AT immune function was modulated by both, diet and parasite infection. In this regard, a similar CD36 receptor expression was induced in DIO and DIO+I groups, but not in LFD+I group. Because parasites were not able to synergize CD36 expression, we consider diet as a key contributing factor for this phenomenon. Thus, we speculate that it may be up-regulated by danger signals like high glucose concentrations, long-chain fatty acids and/or oxLDL[17]. Moreover, CD36-deficiency is strongly associated to increased lipolysis and reduced adipogenesis in mice [38, 39].

Among the chemokines produced from obese VAT, MCP-1 and its receptor CCR-2 appear to be particularly important [8]. It was recently revealed that M2 macrophages are capable to proliferate within CLSs [10]. Of note, intracellular MCP-1 expression was found in CLSs in DIO+I mice.

Importantly, all experimental groups showed a strong VAT leukocyte infiltration compared to LFD group. Previous studies have shown an increase in F4/80+ cells on AT from *T. cruzi* infected mice [15, 29]. In this study, we have characterized for the first time the infiltrating cells in VAT, and found a predominant M2-like phenotype in all groups. A synergism on MCP1+ M2 macrophages was observed in DIO+I compared to DIO and LFD+I groups. We highlight that although predominant M1 phenotype has been reported by our group in *T. cruzi* infected liver tissue [16], ATMs showed a counteracting behavior. Thus, we hypothesized that in VAT, M1 macrophages could have died during the inflammatory process for killing this pathogen, and/or M2 could have proliferated to maintain tissue homeostasis and to promote parasite survival. These findings reinforce the notion that VAT has a clue role as a *T. cruzi* reservoir. Considering that parasite persistence is central for the etiology of Chagas cardiomyopathy, the predominance of M2 macrophages in this tissue could have a high impact on favoring the triggering of myocardial parasitism during the reactivation of infection in chagasic patients. Consistent to the strong cell infiltration in VAT from DIO+I mice, an enhanced lipid peroxidation compared to DIO and LFD+I mice was appreciated at 24 weeks. To our knowledge, this is the first evidence reporting a potentiated oxidative stress in VAT during chronic *T. cruzi* infection in a concomitant obesogenic environment. Recently, Wen et al. (2014) described an oxidative stress process in AT during *T. cruzi* infection [40].

Together, our data suggest that *T. cruzi* could be a potent risk factor for the progression of obesity related-immunometabolic disorders such as diabetes and atherosclerosis.

We highlight that the knowledge of innate immune mechanisms and metabolic dysfunction implicated in obesity and its complications linked to chronic parasite infection may provide new therapeutic strategies for intervention in obesity and globalized Chagas disease.

## MATERIALS AND METHODS

### Ethic statement

Animals were maintained at the Animal Resource Facility of the Facultad de Ciencias Químicas, Universidad Católica de Córdoba (UCC), Argentina, in agreement with the Guide to the Care and Use of Experimental Animals published by the Canadian Council on Animal Care (OLAW Assurance number A5802-01) and approved by the UCC ethic committee.

### Animals and experimental design

C57BL/6J (B6) mice were purchased from the Facultad de Ciencias Veterinarias, Universidad Nacional de la Plata, Argentina and C57BL/10ScNJ mice lacking the Tlr4 gene (Tlr4lps-del mice) were from The Jackson Laboratory, Bar Harbor, ME, USA. Mice were maintained under a standard light cycle (12 h light/dark) and were allowed for free access to water and food. Six-to-eight-week-old male mice were divided randomly and subjected either to a low fat diet (LFD, 3% Kcal fat) or diet-induced obesity treatment (DIO) with medium fat diet (MFD 14% Kcal fat), fructose in water (5%), and a single intraperitoneally (i.p) dose of streptozotocin (STZ, 8 mg/Kg body weight). Infected mice were challenged i.p with 500 blood-derived trypomastigotes (Tulahuen strain) and submitted either to LFD (LFD+I) or DIO treatment (DIO+I). Mice were studied from 4 to 24 weeks. Body weight, waist diameter and VAT content were determined. LFD composition: 3% fat, 26% protein, 53% carbohydrate, 8% crude fiber and 10% total mineral; MFD composition: 14% fat and a similar composition in all respects apart from the total fat content. Bloodstream trypomastigotes were obtained by heart puncture from *T. cruzi*-infected B6 mice at the peak of parasitemia, and parasites were maintained by serial passages from mouse to mouse. Parasites were quantified in tail blood by using a Neubauer chamber, prior lysis with 0.87% ammonium chloride.

### Blood lipids and insulin resistance biomarkers

Blood samples were obtained from cardiac puncture at 4, 12 and 24 weeks. Plasma triglycerides (TG, mg/dL), total cholesterol (TC, mg/dL), glucose (mg/dL) and alanine aminotransferase-activity (ALT, U/L) levels were assessed by enzymatic kits (Roche) with a Hitachi modular P800 auto-analyzer, after 12 hour fasting. Insulin (μU/mL) levels were quantified by insulin RIA kit DPC Coat A Count (Siemens) using Ingetron MODEL MN2200-E after 8 hours fasting and Homeostasis Model of Assessment-Insulin Resistance (HOMA-IR) was calculated as fasting plasma insulin (μU/mL) x fasting blood glucose (mg/dL) / 405. Apolipoprotein B100 (mg/dL) levels were analyzed by an immunoturbidimetry method (Serapak-Bayer) using Modular P800 auto-analyzer, according to manufacturer's instructions. Lipoprotein pattern was assessed based on 3% polyacrylamide slab gel electrophoresis method as described [41], using a TRIS-glycine pH 7.5 buffer.

### Histological analysis

Visceral adipose tissue, heart, abdominal aorta and liver specimens (5μm) were fixed in 4% paraformaldehyde-PBS pH7.4 and embedded in paraffin for hematoxylin and eosin (H&E), and immunofluorescence (IF) studies. For IF, VAT sections were deparaffinized and rehydrated, blocked using PBS containing 2% (w/v) BSA, and incubated with primary antibodies. A specific fluorescence rabbit anti-mouse antibody for monocyte chemotactic protein1 (MCP-1, CCL-2) (Biolegend) was used. After washing, the sections were incubated with FITC-conjugated anti-rabbit antibody (Biolegend). Nuclei were labeled using DNA-binding fluorochrome Hoechst 33258 (2μg/mL). Images were obtained using a Nikon Eclipse TE 2000U equipped with a digital video camera.

### Cytokine assays

ELISA sandwich was performed for quantification of cytokine and adipokine plasma concentrations, as described in our previous study [16]. All regents were purchased from BD Pharmingen and e-Bioscience. Adiponectin and leptin levels were quantified using commercially available ELISA Kits from Abcam and Invitrogen, respectively, and performed according to manufacturer's instructions.

### Western blot analysis

Visceral adipose tissue was lysed in RIPA buffer (50mM Tris-HCl pH7.4, 150mM NaCl, 1%Triton X-100, 0.1%SDS) containing a protease inhibitor cocktail (Sigma-Aldrich) for 30 min, on ice. Equal amounts of protein determined by Bradford assay [42] from each sample were submitted to electrophoresis on 10% SDS-PAGE gels and transferred to nitrocellulose membranes (Millipore, Bedford, MA). A CD36 specific rabbit anti-mouse antibody (Santa Cruz) was used. Horseradish peroxidase-conjugated rabbit anti-mouse immunoglobulin (Santa Cruz) was used to detect specific protein bands using a chemiluminescence system (ECL reagent). For loading control, membranes were incubated with anti-β actin antibody (Santa Cruz). The bands were quantified using Image J software.

### Lipid peroxidation

We evaluated the extent of lipid peroxidation products, i.e. MDA using the TBARS assay as described [43], with some modifications. Briefly, tissue lysates (1:10w/v) were mixed with 0.2ml of 8.1% SDS, 1.5ml of 20% acetic acid pH3.5, and 1.5ml of 0.8% thiobarbituric acid (TBA, Sigma). The mixture was heated at 95°C for 60 min. After cooling on ice, samples were centrifuged at 4000rpm for 10min, and read at 532 nm using a spectrophotometer (HACH, model DR5000). The level of lipid peroxides was expressed in terms of μmol MDA/μg protein.

### Isolation of the SVF of adipose tissue

Mouse epididymal AT was minced and digested for 45min at 37°C with type 2 collagenase (0.8mg/ml; Sigma) in Hanks' Balanced Salt solution (pH7.4). After the addition of 3vol PBS containing 5%FBS and filtration of the digested tissue through nylon mesh (100μm), the filtrate was centrifuged at 200g. The SVF was obtained from the resulting supernatant [44].

### Flow cytometry

The SVF of mouse epididymal AT was prepared as described above. Red blood cells present in this fraction were separated by centrifugation a 500g for 5min, and the remaining cells were suspended in PBS and exposed to FcBLOCK (BD Biosciences) for 20min. One million of SVF cells were washed in ice-cold FACS buffer (PBS-2%FBS) and incubated with fluorochrome labeled antibodies for 20min at 4°C. Different combinations of the following antibodies were used: PECy7-labeled:anti- F4/80, APCCy7-labeled:anti- CD11c, biotin-labeled:anti-CD206 and SA-PECy5. Cells were permeabilized with BD Cytofix/Cytoperm and Perm/Wash (BD Biosciences) for detecting intracellular MCP-1, according to manufacturer's instruction. Then, cells were incubated with PE-labeled antibody for MCP-1. Cells were acquired on FACS Canto II (BD Bioscience).

### Measurement of TG in the liver

Liver tissues (1 g) were homogenized with 1 mL of cold PBS using a hand homogenizer. Homogenates were centrifuged at 5000 rpm for 10min at 4°C. The supernatant was then collected, and TG content was determined using commercial enzymatic kits (Roche) in a Hitachi modular P800 auto-analyzer.

### Myocardial parasite load

For determination of tissue parasitism, genomic DNA was purified from heart of WT LFD+I and DIO+I mice obtained at 4 weeks, using TRIzol reagent and following manufacturer's instructions. Satellite DNA from *T. cruzi* (GenBank AY520036) was quantified by real time PCR using specific Custom Taqman Gene Expression Assay (Applied Biosystem) using the primers and probe sequences described by Piron *et al.* (2007) [45]. A sample containing 1 μg of genomic DNA was amplified. Abundance of satellite DNA from *T. cruzi* was normalized to GAPDH abundance (Taqman Rodent GAPDH Control Reagent, Applied Biosystem) and expressed as arbitrary units.

### Statistical analysis

Results are expressed as means ± SEM. Comparisons among groups were performed using the parametric analysis of variance (one way or two way- ANOVA) followed by a multiple-comparison test (Bonferroni test) using Graph Pad Prism Inc., La Jolla, CA. A *p*-value < 0.05 was considered significant.

## SUPPLEMENTARY MATERIAL FIGURES


