# Supplementary material for: Chronic Trypanosoma cruzi infection potentiates adipose tissue macrophage polarization toward an anti-inflammatory M2 phenotype and contributes to diabetes progression in a diet-induced obesity model
Source: Oncotarget. 2016 Feb 23;7(12):13400–15. doi: 10.18632/oncotarget.7630 (PMC4924650; doi:10.18632/oncotarget.7630)
Supplement: Supplementary file 1 [file oncotarget-07-13400-s001.pdf]

Chronic *Trypanosoma cruzi* infection potentiates adipose tissue macrophage polarization toward an anti-inflammatory M2 phenotype and contributes to diabetes progression in a diet-induced obesity model

Supplementary Material

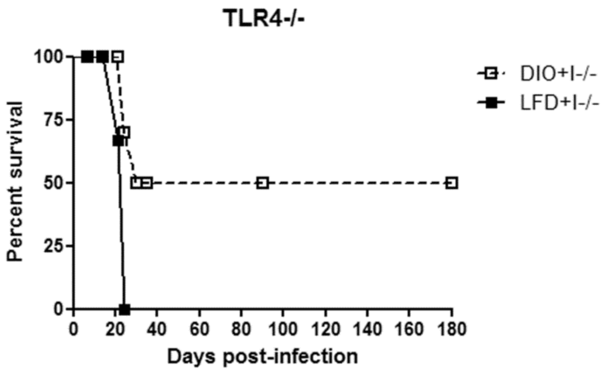

Supplemental Figure 1. Enhanced susceptibility of TLR4-/- LFD+I mice during the acute phase of *T. cruzi* infection. Survival of the TLR4-/- infected groups under both, LFD and DIO treatments was evaluated by a Kaplan-Meier curve.

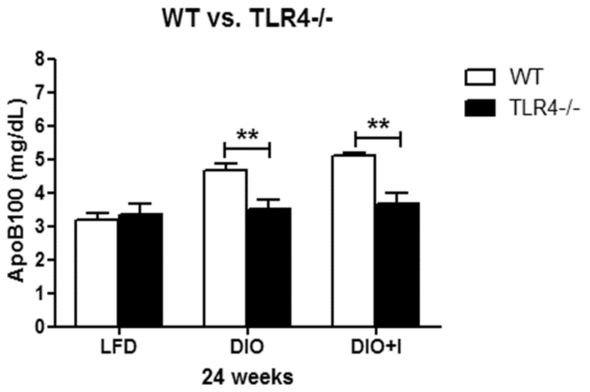

Supplemental Figure 2. TLR4 mediates the increases in apoB100 levels in wild type DIO and DIO+I groups. At 24 weeks apoB100 levels were assayed in TLR4-/- LFD, DIO and DIO+I groups. Results are representative of one experiment of two performed. Data are shown as mean ± SEM of five mice per group. Significant differences were appreciated between WT and TLR4 -/- groups using the two-way ANOVA test: ** $p<0.01$.

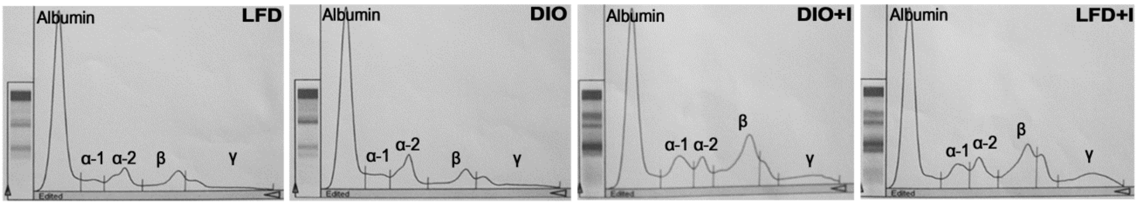

Supplemental Figure 3. Diet and *T. cruzi* infection induce changes in the acute phase protein patterns. Representative images of electrophoretic patterns of all groups are shown at 4 weeks: albumin, α-1 (alpha-1), α-2 (alpha-2), β (beta) and γ (gamma) fractions (from left to right). In rodents, α-1 and α-2 are plasma acute phase proteins.
